# Comparison of Personality among Mothers with Different Parenting Styles 

**Published:** 2018-07

**Authors:** Bita Bahrami, Behrooz Dolatshahi, Abbas Pourshahbaz, Parvaneh Mohammadkhani

**Affiliations:** Department of Clinical Psychology, University of Social Welfare and Rehabilitation Sciences, Tehran, Iran.

**Keywords:** *Authoritarian*, *Authoritative*, *Parenting*, *Parenting Style*, *Personality*, *Permissive*

## Abstract

**Objective:** Mothers have an important role in child- rearing, and maternal personality has theoretically been considered as the most inﬂuential factor determining the parenting style, because it is thought to affect parental behavior. However, the influence of personality on parenting styles has received surprisingly little attention. The aim of the present study was to compare personality components among mothers with authoritative, authoritarian, and permissive parenting styles.

**Method** **:** Using a multistage random cluster sampling method, we selected 8 kindergartens in Tehran. The sample consisted of 270 mothers with preschool children aged 4 to 6 who completed the NEO and Parental Authority Style Questionnaire.

**Results: **Results revealed significant differences among the authoritative, authoritarian, and permissive styles in personality characteristics. There were significant differences between groups in extraversion (f(2,267) = 151.65, p≤0.0001 ), agreeableness (f(2,267)=215.23, p≤0.0001 ), conscientiousness (f(2,267)=336.016, p≤0.0001 ), neuroticism (f(2,267)=1151.1, p≤0.0001 ), and openness to experience (f(2,267)=110.8, p≤0.0001 ).

**Conclusion:** This study revealed the significant role of personality in parenting style.

Parenting styles are patterns for children's training that are created by the normative interaction of parents and how they response to children's behavior (1, 2). Psychologists have identified 4 major parenting styles: authoritative, authoritarian, neglectful/uninvolved, and permissive. Authoritative parenting is a parenting style characterized by high affection and moderate demands of parents. Authoritarian parenting is a strict parenting style characterized by high demands but low responsiveness of parents. They immediately react to misbehaviors of children. (3, 4). 

Permissive parents show much affection, responsiveness, and support to their children, but little control (5). However, neglectful parents provide neither support nor control to their children (6).

Researchers have shown that parenting styles are associated with child development outcomes (3, 4). 

Authoritative parenting has been linked to a number of positive outcomes in children, for example: secure parent-child attachment (7, 8), fewer behavior problems (9), more prosocial behaviors (10), and more positive peer relationships (11). 

Children of authoritarian parenting are apt to possess poor decision- making and low self-esteem, poor social skills and academic competence (12,13), low creativity level, and mental problems such as depression (14) and behavioral issues (15), fear of failure, emotional suppression, and, difficulty in handling negative emotions (16). One of the consequences of permissive parenting for children is a lack of self-control and the development of egocentric behavior (5). Neglectful parenting often leads to more antisocial behavior in children (6).

In the past few decades, Belsky (17) presented a process model for research on the determinants of parenting style. According to this model, parenting behavior is determined by the interplay of 3 different domains: the personal characteristics of the parent (e.g., personality traits and attachment style), the personal characteristics of the child (e.g., temperament), and the social contextual inﬂuences of stress and support (e.g., social support, marital satisfaction) (18). In accordance with Belsky’s model, parental personality was considered the most theoretically substantial cause of parenting because it is thought to affect parental behavior both directly and indirectly (19,20). 

Baumrind and Black (21) found that parents who were controlling, demanding, loving, and communicative had preschool children who were self-controlled, self-reliant, and assertive. They also found that parents who were controlling but detached had unhappy and disaffiliated preschool children, while parents who were relatively warm, but non-controlling and non-demanding had the least self-reliant and self-controlled group of preschool children. Also, some studies have described the relationship between parents’ personality and specific child problem behaviors such as antisocial behaviors and depression (22-25). 

Despite the presumed importance of parental personality and its effect on children’s development and adjustment, its contribution to the quality of parenting has received little attention in empirical research (19, 20). Also, the literature on the associations between personality and parenting styles has not produced the same picture of how personality components relate to parenting behavior, and the results of these researches are incongruent. For example, Losoya et al. (26) found that openness (one component of personality) was associated with more positive support and less negative control, reported by parents, however, Clark et al. (27) found that openness to experience was not associated with observed responsiveness or power assertion with toddlers. Moreover, the literature has examined links between personality and parenting. However, more studies are needed to evaluate how variations in the personality of the nonclinical population relate to differences in parenting (28, 29). Thus, the main aim of the present study was to compare personality components among mothers with different parenting styles in Iran.

## Materials and Methods

This was a cross-sectional study. The statistic community of this study was all mothers in Tehran that had preschool children aged 4 to 6 years old. Based on Cochran’s sample size formula (Variance = 0.5, confidence interval: 0.90%, d = 0.05, Z0.05 = 1.64), a sample size of 270 participants was required. In this study, participants were recruited from 8 kindergartens from 22 districts of Tehran, Iran using multistage random cluster sampling method during 6 months (spring and autumn 2016). Mothers of preschoolers were given verbal and written information about the study. The questionnaires were administered and data were analyzed using SPSS software Version 19 and MANOVA test. 

***Procedures***

This was a causal-comparative study. A total of 8 kindergartens were selected from all kindergartens in Tehran using a multistage sampling method (stratified random cluster) in which each region in Tehran was considered as a stratum and each kindergarten as a cluster. The research questionnaires were distributed among almost 500 mothers of preschool children and 270 were returned, indicating a participation rate of 54%. The study was approved by the Research Ethics Committee of the University of Social Welfare and Rehabilitation Sciences (via approval No.USWR.REC. Dated July 2015), Tehran, Iran.

Trained researcher (first author) explained the purpose and significance of the study to each mother. All participants were informed that participation was voluntary and that their responses would be confidential. After obtaining informed written consent, the research questionnaires package was given to the mothers who agreed to participate in the study. All participants completed the research questionnaires in the presence of researchers, and any questions that they had were answered.

***Measurements***

The NEO-Five Factor Inventory (NEO-FFI) (30) 

The NEO five-factor inventory (NEO-FFI) was developed by Costa & McCrae (30) in Maryland in 1985. This questionnaire consists of 60 questions with five-point Likert scaling (1 = strongly disagree, 2 = disagree, 3 = neutral, 4 = agree, and 5 = strongly agree) and examines 5 scopes of personality traits including openness to experience, conscientiousness, extraversion, agreeableness, and neuroticism. The content validity of NEO Five-Factor Inventory (NEO-FFI) was confirmed by Costa & McCrae (30), and the reliability of neuroticism, extraversion and openness to experience, agreeableness, and conscientiousness was found to be 0.90, 0.78, 0.76, 0.86 and 0.90, respectively. In Iran, the five-factor structure of this questionnaire was generally confirmed by Garousi Farshi et al. (31), and the internal consistency reliability coefficients were reported to be 0.86, 0.73, 0.56, 0.68 and 0.87, respectively, by the measure of Cronbach’s alpha. Agreeableness, neuroticism, conscientiousness, openness to experience, and extraversion had acceptable Cronbach’s alpha coefficients in this study (0.7, 0.87, 0.85, 0.96 and 0.75). 

Parental Authority Questionnaire (PAQ) (32)

In this study, we used a questionnaire relying upon Buri’s (32) PAQ (Parental Authority Questionnaire) developed to measure the parenting styles according to Baumrind’s (21) conceptualization (permissive, authoritarian, authoritative). Buri’s questionnaire consists of 30 items, 10 dedicated to each parenting style. The questionnaire was translated into Persian and validated by Esfandiyari et al. (1995). Reliability and validity of the questionnaire have been confirmed (32). The internal consistency reliability using Cronbach coefficient alpha formula was 0.82 for the authoritarian style. The parental authority scale was based on Likert scale, ranging from 1 (completely disagree) to 5 (completely agree). This grade is calculated by adding the scores of respective questions related to each style. The score for each parenting type can range from 10 to 50. Higher scores in each type indicate that parents are more likely to practice that parenting style with their children. The Cronbach’s Alpha coefficient for permissive, authoritarian, and authoritative styles was 0.85, 0.92 and 0.96, respectively.

## Results

The data were analyzed by SPSS 19. The results of parenting styles analyses showed that 46 mothers had a permissive parenting style (17%), 67 had an authoritarian parenting (24.8%) style, and 157 had an authoritative parenting (58.1%) style. The mean age of mothers was 35.29, SD = 4.46, and it was 5.03, SD =.86 for children. 

Also, descriptive analyzes and MANOVA test were used to examine the hypothesis. The result of Descriptive analyzes data presented in table 1 and the other one at table 2 and 3.

**Table1:** Means and Standard Deviations for Maternal Personality Components in 3 Parenting Styles

**Descriptive Statistics**				
**Parenting Styles**		**Mean**	**Std. Deviation**	**N**
Neuroticism	Permissive	31.88	7.47	46
	Authoritarian	41.69	2.93	67
	Authoritative	9.74	4.51	157
	Total	21.44	14.96	270
Extraversion	Permissive	17.18	3.63	46
	Authoritarian	16.56	3.86	67
	Authoritative	25.25	4.04	157
	Total	21.72	5.72	270
Openness to new experience	Permissive	16.49	3.8	46
	Authoritarian	16.26	3.62	67
	Authoritative	23.29	3.82	157
	Total	20.39	5.08	270
Agreeableness	Permissive	14.36	2.99	46
	Authoritarian	12.76	3.03	67
	Authoritative	22.95	4.18	157
	Total	18.96	6.03	270
Conscientious	Permissive	10.51	5.17	46
	Authoritarian	32.75	5.23	67
	Authoritative	20.44	5.67	157
	Total	25.9	10.25	270

According to Table 1, authoritative mothers had the lowest mean in neuroticism and the highest in extraversion, openness to experience, and agreeableness. The authoritarian mothers had the lowest mean in extraversion, openness to experience, and agreeableness and the highest mean in neuroticism and conscientiousness.

**Table2:** MANOVA test for Comparison of Maternal Personality Components in 3 Parenting Styles

**Source**	**Dependent Variable**		**Type III Sum ** **of Squares**	**Df**	**Mean ** **Square**	**F**	**Sig.**	**Partial Eta ** **Squared**
Parenting style	Neuroticism		53995.78	2	26997.89	1151.1	0.00	0.89
	Extraversion		4691.46	2	2345.73	151.65	0.000	0.53
		Openness	3158.83	2	1579.41	110.8	0.000	0.45
		Agreeable	6047.92	2	3023.96	215.23	0.000	0.61
		Conscientiousness	20254.78	2	10127.39	336.01	0.000	0.71

According to Table 2, there were significant differences between groups in extraversion (f(2,267) = 151.65, p≤0.0001 ), agreeableness (f(2,267)=215.23, p≤0.0001 ), conscientiousness (f(2,267)=336.016, p≤0.0001 ), neuroticism (f(2,267)=1151.1, p≤0.0001 ), and openness to experience (f(2,267)=110.8, p≤0.0001 ). The Bonferroni test was administered to make dyadic comparisons.

**Table3:** Dyadic Comparisons of Groups in Personality Components (Bonferroni Test)

**Dependent ** **Variable**	**(I) Parenting ** **style**	**(J) Parenting** **Style**	**Mean Difference (I-** **J)**	**Std. ** **Error**	**Sig.**
Neuroticism	Permissive	Authoritarian	-9.8^*^	0.92	0.000
		Authoritative	22.14^*^	0.81	0.000
	Authoritative	Authoritarian	-31.95^*^	0.7	0.000
			
Extraversion	Permissive	Authoritative	0.61	0.75	0.689
		Authoritative	-8.07^*^	0.65	0.000
	Authoritative	Authoritarian	8.69^*^	0.57	0.000
			
Openness	Permissive	Authoritarian	0.22	0.72	0.948
		Authoritative	-6.79^*^	0.63	0.000
	Authoritative	Authoritarian	7.02^*^	0.55	0.000
			
Agreeableness	Permissive	Authoritarian	1.59	0.71	0.068
		Authoritative	-8.59^*^	0.62	0.000
	Authoritative	Authoritarian	10.18^*^	0.54	0.000
			
Consciousness	Permissive	Authoritarian	-22.23^*^	1.05	0.000
		Authoritative	12.3^*^	0.92	0.000
	Authoritative	Authoritarian	-9.93^*^	0.8	0.000
			

According to this table, the differences between groups were significant (p≤0.0001) in all the personality components, except for the differences between the permissive and authoritarian styles in extraversion (MD =.61, P>0.05), openness to new experience (MD = -.22, P>0.05), and agreeableness (MD=-1.59, P>0.05).

## Discussion

The present study examined the role of parents’ personality in parenting style by comparing personality characteristics in Iranian mothers who had different parenting styles. Findings supported the effect of personality on parenting styles. Our finding showed that authoritative mothers had high scores in extraversion, openness to experience, and conscientiousness, and a low score in neuroticism, but authoritarian and permissive mothers had high scores in neuroticism.

 Previous studies indicated that mothers who were high in authoritarian and permissive parenting had high scores in neuroticism. Neuroticism has received the most attention in the literature with regards to parenting manners (33). People high in neuroticism tend to become easily nervous, tense, anxious, and lack emotional stability. These characteristics are likely to interfere with sensitive parenting because parents high in neuroticism may become focused on themselves, which may not allow them to be sensitive to their children’s needs. The result of the present study showed that authoritative parents have a low score on this characteristic. So, they can have a moderate control over their children.

Also, there was a significant difference between groups in extraversion. Authoritative mothers had a high score in extraversion, but permissive and authoritarian mothers obtained low scores. Mothers high on extraversion may be expected to be more sensitive to their toddlers’ cues because people high on extraversion tend to be energetic, affectionate, talkative, and optimistic. Extraverted individuals love social interactions, which could include interactions with children. Smith et al. (9) found that extraversion was correlated with parental reports of positive emotional expressiveness towards their 24-month old children (34).

Rothbart, Ahadi, and Evans (35) found that individuals high in extraversion were high in adult temperament factors including activity level, pleasure reactivity, sociability, and high-intensity pleasure. The results of the present study showed that authoritative parents have a high score on this characteristic. So, they can show more affection and love to their children.

Another finding of this study indicated that agreeableness is higher in authoritative mothers than in permissive and authoritarian ones. Agreeableness is the desire to maintain positive social relationships and act in ways that promote those relationships. Graziano et al. have found that students and adolescents high in agreeableness were less competitive and more likely to do techniques focused on the agreement. These findings support the opinion that the personality dimension of agreeableness develops behaviors to support and enhance social interactions. Individuals with higher levels of agreeableness would exhibit more sensitive and less intrusive parenting, as they are better able to follow the cues of others and seek to sustain welcome interactions with their children. This has been substantiated in the research, as mothers with higher levels of agreeableness were found to show more sensitive parenting behaviors and more positive emotional expressions during free play with their 18-month old toddlers (9). Agreeableness was negatively correlated with parental reports of negative mood and observed negative affect with toddlers. Researchers have found that disagreeableness was positively correlated with power assertion and detachment and negatively correlated with sensitivity and warmth, as assessed using videotaped interaction (28). In addition, it has been found that mothers who reported lower levels of agreeableness were observed to be more detached from their 9-month old infants (19). Additionally, empathy may be a facet of agreeableness (36) and could facilitate a parent’s ability to perceive children’s signals and respond sensitively (34). Inconsistent with these findings, our authoritative mothers in the sample group with high agreeableness could maintain positive relationships with their preschool children.

Also, the results revealed that openness was higher in authoritative mothers than in permissive and authoritarian ones. In general, those people who are open to experience are high on imagination, intellectual interests, and enjoyment of new experiences. People who are open to experience may be more sensitive as parents. Inconsistent with this study’s finding, Losoya et al. (26) found that openness to experience was related to more positive support and less negative control in parents of school-aged children. Also, Prinzie et al. (37) found positive relationships between openness and non-intrusive parenting, sensitivity, and warmth. It was thought that parents higher in openness are likely to view the autonomy of their children in a positive light rather than an offense to parental authority (34, 37). According to these explanations, our finding on the high score of authoritative mothers in openness to experience was not surprising.

Finally, findings revealed that authoritarian and authoritative mothers had the highest and a high scores in conscientiousness, respectively. Conscientious people have a high score in constraint, control, responsibility, dependability, and adhering to rules and norms. Clark (27) found that high conscientiousness was associated with more maternal responsiveness and support. Also, high levels of conscientiousness have been found to be associated with more positive support and less negative control reported by parents of children. These findings support the opinion that organized and purposeful personality traits may facilitate authoritative parenting. Whereas extremely high levels of conscientiousness may place too many demands on young children because it develops standards in parenting rules (33), and thus may be linked with intrusive or over-controlling behaviors in authoritarian parents.

## Limitation

First, there are many factors such as attachment style, social support, marital satisfaction, work stress, child’s temperament, family socio economic status (SES), and ethnicity (7) that can affect parenting style. Although the present study could not investigate them, they should be investigated further. Second, this study was administered to mothers only, and conducting a similar study on fathers is also recommended. Also, we solely used self-report measures, and thus future studies should include information gathered via multiple methods (for example, observational methods) to ensure the validity of the study. Finally, small sample size and not controlling the sample’s social class could restrict the generalization of our findings, so replicating this study with larger sample sizes from different social class groups is highly suggested.

## Conclusion

This study revealed the significant role of personality in parenting style. Our study improves the understanding of the relationship between personality and parenting styles. According to our finding, the most beneficial parent (with authoritative parenting) would be one who is high in extraversion, openness to experience, and conscientiousness, and low in neuroticism.
